# A novel protein-coding ORF72.2 gene was identified from Marek's disease virus strain CVI988

**DOI:** 10.1186/1743-422X-7-371

**Published:** 2010-12-23

**Authors:** Mingxing Tian, Yang Zhao, Min Shi, Yan Lin, Nianli Zou, Ping Liu, Xintian Wen, Sanjie Cao, Yong Huang

**Affiliations:** 1 College of Veterinary Medicine, Sichuan Agricultural University, Ya'an, Sichuan, 625014, PR China; 2 Key Laboratory of Animal Disease and Human Health of Sichuan Province, Sichuan Agricultural University, Ya'an, Sichuan, 625014, PR China

## Abstract

Marek's disease is a highly contagious disease of poultry characterized by rapid-on set of T-cell lymphomas, which is caused by Marek's disease virus (MDV), but its pathogenic mechanism is still not very clear. Recently, some new progress were achieved in molecular character of MDV. Along with the genomic sequencing of MDV serotype 1, some novel open reading frames (ORFs) were predicted, and ORF72.2 was one of them which have no homologues in other MDV serotypes or in other alphaherpesvirus. In the study, ORF72.2 was firstly identified as a protein-coding gene by the method of reverse transcription polymerase chain reaction (RT-PCR), western blotting and indirect immunofluorescence assay. This study paved the way to conduct further studies to determine whether ORF72.2 plays a role in MDV replication and pathogenicity.

## Findings

Marek's disease (MD) is a highly contagious disease of poultry characterized by mononuclear cellular infiltrates in peripheral nerves and various other organs and tissues including iris and skin. The disease has a worldwide distribution and remains a major concern for the poultry industry even though vaccines are used widely today [1]. The causative agent of the disease is Gallid herpesvirus 2, which is also called Marek's disease virus (MDV) serotype 1. This virus is an alphaherpesvirus of the genus Mardivirus [2], which also includes the antigen-related Meleagrid herpesvirus 1 (HVT), a strain used widely as a vaccine against MD since the late 1960s [3,4], as well as Gallid herpesvirus 3 (MDV-2), which includes apathogenic strains some of which are used as live vaccines against MD [5].

The complete genomic sequence of the MDV-1 vaccine strain CVI988 was determined in 2007 which consisting of 178311 bp with an overall gene organization identical to that of the oncogenic strains such as GA, RB1B, Md5, Md11, 584A. The genome of CVI988-BAC contains over 478 ORFs encoding proteins with more than 50 amino acid (aa) residues [6]. Among the ORFs, the putative functions of some ORFs were predicted based on the comparison with homologous genes of other alphaherpesvirus whose functions had already been well known [7,8], however, some ORFs are found to have no homologues in other alphaherpesvirus. ORF72.2 in vaccine strain CVI988 was one of those ORFs and consists of 621 nucleotides, encoding 206 aa residues. In this study, the ORF72.2 protein was firstly indentified, expressed and localized in cell by western blotting and immunofluorescence assay, which laid the foundation for the study of pathogenic mechanism of MDV.

**Figure 1:**
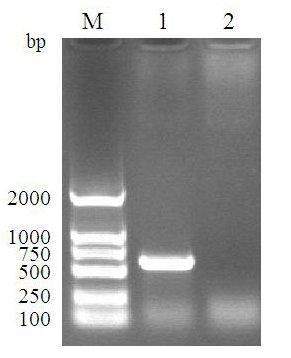
**Amplification results of ORF72.2 gene by RT-PCR**. M: DNA Marker DL2000; Lane 1: RT-PCR result of ORF72.2 gene from MDV-infected CEFs; Lane 3: RT-PCR result of ORF72.2 gene from normal CEFs.

The MDV CVI988 strain used in this study was grown in chicken embryo fibroblast (CEF) cells. Cell cultures were maintained in modified Eagle's medium (MEM) supplemented with 10% fetal bovine serum (FBS), 0.22% NaHCO3, 100 IU/ml penicillin and streptomycin [9]. The RNA was extracted using RNAprep pure Cell Kit (TIANGEN, Beijing, China) from normal CEFs and MDV-infected CEFs at 24 h, 48 h, 72 h, 96 h post-infection. Then a pair of primers were designed based on the bioinformatics analysis (Primer Premier 5.0 software) of the ORF72.2 gene and used to amplify a 590 base pair (bp) fragment spanning nt 22 through 612 of ORF72.2 gene, and the amplification was performed with reverse transcription polymerase chain reaction (RT-PCR) (Figure 1). The sequences of the primers used for this purpose were R1: 5'-CCGGAATTCCCTGATACTGCTAAGAGATCAC-3' with *Eco*RI site (underlined) and R2: 5'-CCCAAGCTTCGTATACAGCCGAACATAAT-3' with *Hin*d III site (underlined). The result showed that the ORF72.2 gene was successfully amplified from MDV-infected CEFs by RT-PCR at all time points tested, which suggested that the ORF72.2 gene was transcribed persistently in middle and late time of viral infection.

**Figure 2:**
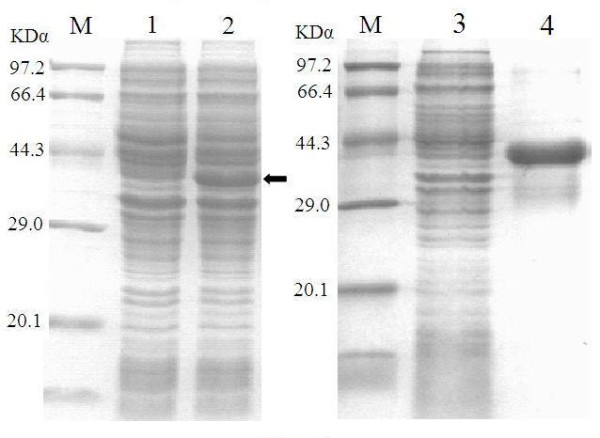
**Results of expression and purification of recombinant ORF72.2 protein**. M: protein marker; Lane 1: the total cell proteins of uninduced BL21 containing recombinant plasmid; Lane 2: the total cell proteins of induced BL21 containing recombinant plasmid; Lane 3: the supernatant of cell lysate of induced BL21 containing recombinant plasmid; Lane 4: purified fusion protein.

The amplified product was cloned into pET32 (+) plasmid to get a recombinant plasmid named pET32-ORF72.2. Escherichia coli BL21 (DE3) were transformed with the recombinant plasmid, and protein expression was induced with 1 mM IPTG at 37°C for 4 h. The bacterial proteins were analyzed by 12% SDS-PAGE under denaturing conditions. Protein bands were visualized after staining with 0.1% Coomassie blue R250, and the protein concentration was determined using program BandScan 5.0 [10]. The recombinant ORF72.2 protein with molecular weight of about 41 KDa by SDS-PAGE analysis was successfully expressed in the transformed cells (Figure 2).

The expressed recombinant ORF72.2 protein was trapped in inclusion bodies. The cells were harvested by centrifugation and resuspended in Phosphate-Buffered Saline (PBS) (pH 8.0) containing lysozyme (0.1 mg/mL) by 1/10 (v/v), after ice bathing for 30 min, the suspension were sonicated and centrifuged at 12000 g for 10 min. The pellets were homogenated and washed with washing buffer [50 mM Tris-HCl (pH 8.0), 1 mM EDTA, 0.2% Triton X-100, 2 M urea] for three times at 10 min/times and centrifugated at 12000 g for 10 min. The pellets were dissolved by denaturation buffer [50 mM Tris-HCl (pH 8.0), 2 mM 2-mercaptoethanol, 8 M urea] and supernatant were collected after centrifugation. The supernatant was treated with renaturation buffer [50 mM Tris-HCl (pH 8.0), 0.1 mM oxidized glutathione, 1 mM reduced glutathione, 0.5 M urea] for overnight at 4°C and filtrated through 0.45 uM filtration membrane. Then the solution was purified on a column packed with Ni-NTA His Bind superflow according to the manufacture's instruction (Merck, Darmstadt, Germany). Bound protein fractions were pooled, dialyzed and concentrated, and the protein expression yield was analyzed by Bradford assay [11]. For the purified fusion protein, a single objective band was detected by SDS-PAGE (Figure 2).

For production of chicken antiserum against MDV, 1-day-old specific pathogen-free (SPF) chicken were injected intraperitoneally (i.p) with 0.2 ml of MDV CVI988 (Intervet) containing 4000 plague forming unit (PFU), and booster injection was done at days 10 using the same method [12]. Two weeks after immunization, the blood was harvested from jugular vein and the antiserum was isolated. The serum of non-immunized chicken was also isolated as control.

For production of rabbit antiserum against recombinant ORF72.2 protein, New Zealand white rabbits were first immunized intradermally with 1 mg purified recombinant protein mixed with an equal volume of complete Freund adjuvant (Sigma, Shanghai, China) and with purified recombinant protein mixed with an equal volume of incomplete Freund adjuvant on days 14 and 28 later as booster injection, respectively. The serum was collected at 10 days after the final booster injection. The antiserum was purified using ammonium sulfate precipitation and High-Q anion-exchange chromatography [13].

**Figure 3:**
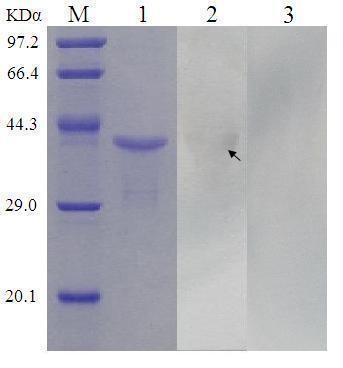
**Demonstration of antibodies against ORF72.2 protein in serum of MDV-infected chickens by Western blotting**. M: protein marker; Lane 1: purified fusion protein; Lane 2: Western-blot result of purified recombinant protein with chicken antiserum against MDV; Lane3: Western-blot result of purified recombinant protein with serum from normal chickens.

To testify indirectly whether the ORF72.2 protein can be expressed in chickens after MDV CVI988 infection, the chicken antiserum against MDV produced above was used to make a western-blot. Firstly, purified recombinant proteins were separated by SDS-PAGE, and then the proteins were transferred to Polybinylidene Difluoride (PVDF) membrane with 0.25 uM pore size (Millipore Corp., USA) at 15 V for 1.5 h. Then the membrane was blocked for 90 min with milk buffer [20 mM Tris-HCl (pH8.0), 150 mM NaCl, 0.05% Tween 20, 5% skinned dry milk] at 37°C and washed with Tris-Buffered Saline with Tween 20 (TBST) buffer [20 mM Tris-HCl (pH8.0), 150 mM NaCl, 0.05% Tween20] for three times. Then the membrane was incubated with chicken antiserum diluted 1:50 in 0.1% Bovine Serum Albumin (BSA)/PBS for 60 min at 37°C and washed with TBST, and then incubated with horseradish peroxidase (HRP)-labeled rabbit-anti-chicken IgG (Zhongshan Goldenbridge Biotechology co., Ltd, Beijing, China) for 60 min at 37°C. Target proteins were visualized using 3, 3'-Diaminobezidine (DAB) (TIANGEN, Beijing, China). The result of western blotting showed the fusion protein can react positively with chicken antiserum against MDV (Figure 3), which proved that the ORF72.2 protein can be expressed in chickens after MDV infection and an immune response can be induced by natural ORF72.2 protein, but band line of western blotting was light, implying the expression level or immunogenicity of natural ORF72.2 protein in chicken after MDVs infection is weak.

**Figure 4:**
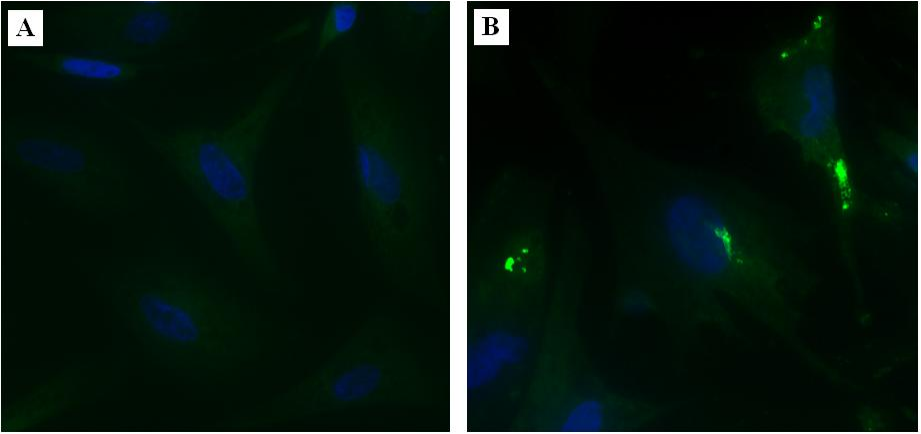
**Detection of ORF72.2 protein by immunofluorescence assay in non-infected CEFs (A) and MDV-infected CEFs (B)**. CEFs were incubated with rabbit antiserum against recombinant ORF72.2 protein, and subsequently stained with fluorescein isothiocyanate (FITC)-conjugated sheep-anti-rabbit IgG. Nuclear were counterstained with DAPI (blue).

To further confirm the existence of ORF72.2 protein in MDV-infected CEFs, indirect immunofluorescence studies were performed with the rabbit antiserum against recombinant ORF72.2 protein produced above. CEFs were mock-infected or infected with MDV and were collected at 72 h post-infection when cytopathogenic effect (CPE) had just appeared. Cells on coverslips were fixed with 4% cold paraformaldehyde for overnight at 4°C and treated with 3% BSA to block the nonspecific staining, and then permeabilized with 0.2% (v/v) TrionX-100 in PBS for 20 min at room temperature. The samples were incubated with rabbit antiserum against recombinant ORF72.2 protein diluted in 1:100 for overnight at 4°C and washed with TBST for three times, and then incubated with fluorescein isothiocyanate (FITC)-conjugated sheep-anti-rabbit IgG (Zhongshan Goldenbridge Biotechology co., Ltd, Beijing, China) for 1 h at 37°C [14,15]. The result showed that specific fluorescence appeared in cytoplasm and nuclear membrane region of infected cells at 72 h post-infection (Figure 4).

In this study, ORF72.2 gene was identified from MDV CVI988 in the level of transcription and expression. Firstly, mRNA was extracted from CEFs infected by CVI988 using RT-PCR, which proved that ORF72.2 gene is successfully transcribed in infected cells. Secondly, the highly antigenic region of ORF72.2 gene was successfully cloned and expressed in an E. coli pET32 (+) expression system. Thirdly, the existence of antibodies against natural ORF72.2 protein in chickens after MDV infection was verified by western blotting using recombinant ORF72.2 protein as the antigen. Meanwhile, the existence of ORF72.2 protein in MDV-infected CEFs was further verified by immunofluorescence assay using rabbit antiserum against recombinant protein. Generally, natural ORF72.2 protein was found in MDV infected cells and natural antibodies against ORF72.2 protein can be detected in chicken after MDV infection, all this proved that ORF72.2 gene is really a novel protein-coding gene of MDV.

In addition, bioinformatics analysis of the complete genomic sequence of MDV revealed that ORF72.2 gene existed both in mild virulent MDVs (such as CU-2) and high virulent MDVs (such as GA, RB1B, MD5, MD11, 584A), but it was only found in MDV serotype 1 and its homologous gene was not identified in MDV serotype 2 and 3 or in other alphaherpesvirus. Deduced amino acid sequence of ORF72.2 was searched for the existence of protein motifs in the PROSITE pattern database and the function of this protein was searched too, but no available database and protein motifs could be found.

In conclusion, a novel protein encoded by ORF72.2 gene was identified and verified by method of western blotting and indirect immunofluorescence assay (IFA), however, it was not really clear whether this protein is important to the replication and pathogenicity of MDV, further study should be undertaken in the following study.

## Competing interests

The authors declare that they have no competing interests.

## Authors' contributions

MT: Study design, performed the experiments, interpreted the data and wrote the manuscript. YZ, MS, YL, NZ, PL, XW, SC and YH: helped in experiments and drafting the manuscript. All authors read and approved the final manuscript.

## References

[B1] Saif YM Barnes HJ Diseases of poultry 2008 12 Ames, Iowa: Blackwell Pub. Professional 452 514

[B2] Berger P Adams M Barnett O Brunt A Hammond J Hill J Jordan R Kashiwazaki S Rybicki E Spence N Virus Taxonomy. Eighth Report of the International Committee on Taxonomy of Viruses 2005 Academic Press

[B3] Witter RL Protection by attenuated and polyvalent vaccines against highly virulent strains of Marek's disease virus Avian Pathol 1982 11 49 62 10.1080/03079458208436081 18770172

[B4] Kawamura H King DJ Anderson DP A herpesvirus isolated from kidney cell culture of normal turkeys Avian Dis 1969 13 853 863 10.2307/1588592 4902778

[B5] Schat KA Calnek BW Characterization of an apparently nononcogenic Marek's disease virus J Natl Cancer Inst 1978 60 1075 1082 76680 10.1093/jnci/60.5.1075

[B6] Spatz S Petherbridge L Zhao Y Nair V Comparative full-length sequence analysis of oncogenic and vaccine (Rispens) strains of Marek's disease virus Journal of General Virology 2007 88 1080 10.1099/vir.0.82600-0 17374751

[B7] Niikura M Liu HC Dodgson JB Cheng HH A comprehensive screen for chicken proteins that interact with proteins unique to virulent strains of Marek's disease virus Poult Sci 2004 83 1117 1123 15285502 10.1093/ps/83.7.1117

[B8] Nishiyama Y Herpes simplex virus gene products: the accessories reflect her lifestyle well Rev Med Virol 2004 14 33 46 10.1002/rmv.409 14716690

[B9] Imai K Yuasa N Kobayashp S Nakamura K Tsukamoto K Hihara H Isolation of Marek's disease virus from Japanese quail with lymphoproliferative disease Avian Pathol 1990 19 119 129 10.1080/03079459008418661 18679919

[B10] Gunasekera RS Damodaran H Rajakarunanayake Y Hyland K The Significance of Linearity of Quantities in Electrophoresed and Blotted Materials Demonstrated by BandScan - an Analytical Program Proceedings of the 2005 IEEE Computational Systems Bioinformatics Conference - Workshops 2005 IEEE Computer Society 277 282 277-282full_text

[B11] Bradford MM A rapid and sensitive method for the quantitation of microgram quantities of protein utilizing the principle of protein-dye binding Anal Biochem 1976 72 248 254 10.1016/0003-2697(76)90527-3 942051

[B12] Liang P Comparative studies on the vaccination and revaccination of Marek's disease 2005 Yangzhou University, Veterinary College

[B13] McGuire JM Douglas M Smith KD The resolution of the neutral N-linked oligosaccharides of IgG by high pH anion-exchange chromatography Carbohydr Res 1996 292 1 9 8870235 10.1016/s0008-6215(96)91015-0

[B14] Xie W Cheng A Wang M Chang H Zhu D Luo Q Jia R Chen X Expression and characterization of the UL31 protein from duck enteritis virus Virol J 2009 6 19 10.1186/1743-422X-6-19 19208242 PMC2661054

[B15] Xiang J Ma G Zhang S Cheng A Wang M Zhu D Jia R Luo Q Chen Z Chen X Expression and intracellular localization of duck enteritis virus pUL38 protein Virol J 2010 7 162 10.1186/1743-422X-7-162 20637115 PMC2918563

